# The Prevalence of Latent *Mycobacterium tuberculosis* Infection Based on an Interferon-γ Release Assay: A Cross-Sectional Survey among Urban Adults in Mwanza, Tanzania

**DOI:** 10.1371/journal.pone.0064008

**Published:** 2013-05-21

**Authors:** Andreas V. Jensen, Lotte Jensen, Daniel Faurholt-Jepsen, Martine G. Aabye, George Praygod, Jeremiah Kidola, Maria Faurholt-Jepsen, John Changalucha, Nyagosya Range, Henrik Krarup, Henrik Friis, Aase B. Andersen

**Affiliations:** 1 Department of Infectious Diseases, Odense University Hospital, Odense, Denmark; 2 Department of Nutrition, Exercise and Sports, Faculty of Science, University of Copenhagen, Copenhagen, Denmark; 3 Clinical Research Centre, University of Copenhagen, Hvidovre Hospital, Copenhagen, Denmark; 4 Mwanza Research Centre, National Institute for Medical Research, Mwanza, Tanzania; 5 Muhimbili Research Centre, National Institute for Medical Research, Dar Es Salaam, Tanzania; 6 Department of Clinical Biochemistry, Aalborg University Hospital, Aalborg, Denmark; McGill University, Canada

## Abstract

**Introduction:**

One third of the world’s population is estimated to be latently infected with *Mycobacterium tuberculosis* (LTBI). Surveys of LTBI are rarely performed in resource poor TB high endemic countries like Tanzania although low-income countries harbor the largest burden of the worlds LTBI. The primary objective was to estimate the prevalence of LTBI in household contacts of pulmonary TB cases and a group of apparently healthy neighborhood controls in an urban setting of such a country. Secondly we assessed potential impact of LTBI on inflammation by quantitating circulating levels of an acute phase reactant: alpha-1-acid glycoprotein (AGP) in neighborhood controls.

**Methods:**

The study was nested within the framework of two nutrition studies among TB patients in Mwanza, Tanzania. Household contacts- and neighborhood controls were invited to participate. The study involved a questionnaire, BMI determination and blood samples to measure AGP, HIV testing and a Quantiferon Gold In tube (QFN-IT) test to detect signs of LTBI.

**Results:**

245 household contacts and 192 neighborhood controls had available QFN-IT data. Among household contacts, the proportion of QFT-IT positive was 59% compared to 41% in the neighborhood controls (p = 0.001). In a linear regression model adjusted for sex, age, CD4 and HIV, a QFT-IT positive test was associated with a 10% higher level of alpha-1-acid glycoprotein(AGP) (10^B^ 1.10, 95% CI 1.01; 1.20, p = 0.03), compared to individuals with a QFT-IT negative test.

**Conclusion:**

LTBI is highly prevalent among apparently healthy urban Tanzanians even without known exposure to TB in the household. LTBI was found to be associated with elevated levels of AGP. The implications of this observation merit further studies.

## Introduction

One third of the world’s population is estimated to be latently infected with *Mycobacterium tuberculosis* (LTBI) [1]. From this immense pool, around 200 million people will eventually progress to active tuberculosis (TB), and in this way fuel the TB epidemic [2]. Several factors are associated with the progression from LTBI to active TB such as malnutrition, smoking, diabetes, and compromised immunity with HIV being one of the strongest risk factors [3].

The Tuberculin Skin Test (TST), developed by R. Koch in 1890 and refined by C. Mantoux in 1907, is widely used to detect LTBI. However, the TST has some important limitations such as low specificity among Bacilli Calmette-Guérin (BCG) vaccinated individuals [4] and a relatively low sensitivity in immune compromised individuals [5].

During the recent decade new immunological tests for diagnosing *M. tuberculosis* infection have been developed. The Interferon-γ (IFN-γ) release assays (IGRA) are in vitro assays measuring the IFN-γ release of sensitized T-cells after exposure to *M. tuberculosis* antigens. The *M. tuberculosis* antigens used are encoded in the Region of Difference-1, a part of the genome which is absent in all BCG vaccine strains as well as in most non-tuberculous mycobacteria [6]. Latent TB infection is often perceived as a completely inert “bystander” infection, with no influence on the host. However, we know that a proportion of LTBI at some point reactivate and develop active disease, and this may be preceded by a “fight” between host and microorganism leading to inflammatory responses that could have long term impact on the infected person. The acute phase reactant alpha-1-acid glycoprotein (AGP) has previously been associated with active TB [7], [8] and it might be a marker of inflammation in regards to LTBI. The primary objective was to estimate the prevalence of LTBI in household contacts of pulmonary TB cases and a group of apparently healthy neighborhood controls in an urban setting in a TB high endemic country. The secondary objective was to detect signs of inflammation in persons with LTBI by measuring AGP.

## Methods

### Ethics Statement

Ethical permission was obtained from the Medical Research Coordinating committee of the National Institute for Medical Research (NIMR) in Tanzania. Study approval was given by The Danish Central Medical Ethics Committee. Written and oral information was presented to all eligible participants by the health staff before written informed consent was obtained. Written consent was obtained from parents/legal guardians of any participants between 15–18 years of age. Pre-HIV test counseling was compulsory, and post-HIV-test counseling was offered to all HIV-positive participants. Patients diagnosed with HIV were referred for further management.

### Study Environment

The present study was nested within the framework of two nutrition studies among newly diagnosed TB patients [9], [10], from where a number of non-TB individuals were invited to participate as controls. The present study was conducted from January 2007 to May 2008 at four TB clinics in Tanzania’s second largest city Mwanza, and it is based on data from the non-TB participants. All non-TB participants recruited within this period (n = 455) were invited to participate in the present study. The TB clinics cover most of the city area including periurban districts, thus the participants were recruited from a wide city area with varying socio-demography. Mwanza city holds approximately half a million inhabitants, and is located on the southern shores of Lake Victoria. Mwanza city is administratively divided into 21 wards, 208 sub-wards and about 500 streets. Each street is further divided into an informal communal cell with 10–20 households (ten-cell), headed by a ten-cell leader.

### Recruitment and Eligibility

To serve as non-TB controls for the TB study, individuals from the neighborhood of TB patients as well as individuals from the household of TB patients were invited to participate in the present study. To be eligible, only adult (>15 years) participants were invited. Furthermore, the participants were not eligible if they had any history of TB, evidence of active TB (cough, intermittent fevers, excessive night sweating in the past two weeks, unexplained weight loss in the past month), were pregnant or lactating, suffering from other severe diseases, or non-residents of Mwanza City. The participants HIV status did not influence the eligibility.

#### The neighborhood controls

The TB cases participating in the nutrition studies [9], [10] were asked to provide detailed contact information about their ten-cell leader, and in co-operation with the ten-cell leader a complete list of individuals from the same ten-cell, with the same sex and similar age (+/−5 years) as the case, was made. From the list one individual was randomly selected by drawing a number from a hat. The number corresponds to a name in the list given by the ten cell leader. The individual was invited to participate as neighborhood control. None of the neighborhood controls were household contacts of a TB patient. If the invited control was not eligible another was randomly selected from the list. The recruitment was performed in weekdays during daytime between 8 am and 5 pm.

#### The household contacts

The TB patients from which the neighborhood controls were invited also provided acceptance for a staff member to visit the household of the TB patient. Within the household one eligible individual was randomly selected using same method as above mentioned. The recruitment was performed in weekdays during daytime between 8 am and 5 pm.

### Study Assessment and Measurement

At recruitment all individuals were interviewed by a health care worker using a questionnaire about socio-demographical information.

Weight and height of the participants were measured (nearest 0.1 kg and 0.1 cm), from which Body mass index (bmi) (kg/m^2^) was derived. For each participant the presence or absence of a BCG scar was determined by a trained nurse at the TB clinic.

### Laboratory Analyses

Blood samples were collected in heparinized tubes (Lithium heparinized plasma containers 4 ml, Becton Dickinson (BD) Vacutainer®, BD Brøndby, DK) and transported to the central study laboratory within 8 hours. HIV testing was performed in duplicates, using rapid tests Determine HIV 1/2 (Inverness Medical Innovations, Inc.) and Capillus HIV-1/HIV-2 (Trinity Biotech Plc.). If discordance was found between the two tests a confirmatory ELISA test was performed (OrganonUniform II, Organon Teknia, NL).

Cluster of differentiation 4 (CD4) count was obtained by flow cytometry after CD4 immuno-flourochrome staining of the leucocytes using the Partec Facs machine (GmbH. Münster, FRG). Also hemoglobin levels (g/dL), white blood cell (WBC) (10^9^/L) counts (including differentials), were analyzed at the central laboratory.

For neighborhood controls only, serum concentrations of the acute phase reactant alpha-1-acid glycoprotein (AGP) were determined at the Department of Clinical Biochemistry, Aalborg University Hospital, DK.

### TB Diagnostics Based on an IGRA

#### QuantiFERON®-TB gold In-tube method (QFT-IT)

Blood was collected from study participants in heparinized tubes (Lithium heparinized plasma containers 4 ml, BD Vacutainer®, BD, Brøndby, DK) in each of the four main study centers and transported to the study laboratory within 8 hours. One ml of blood was transferred to each of the three QFT-IT test blood collection tubes pre-coated with (i) the TB-specific antigens, (ii) a phytohemagglutinin (PHA) positive control (Mitogen) and (iii) a negative (Nil) control tube, respectively. The samples were shaken to ensure proper mixing of blood with contents and incubated upright at temperature 37°C for 20–24 hours. They were centrifuged at 3,000×g for 10 minutes and plasma was harvested and collected in Nunc® cryotubes (Nunc, Roskilde, DK) and stored frozen at –80°C. Plasma samples were transported on dry ice from Tanzania to Denmark, where ELISA testing was performed as described by the manufacturer (www.cellestis.com).

ELISA readings were transferred to the QFT-Gold v.2.50 Software giving final QFT-IT test results. The QFT-IT result was graded as positive if the IFN-γ in plasma of the antigen-stimulated blood was >0.35 IU/ml after subtracting the cytokine concentration of unstimulated plasma and the stimulation index (IFN–γ concentration in plasma of antigen stimulated blood divided by IFN–γ concentration of unstimulated blood) was ≥1.25 regardless of the mitogen stimulated IFN–γ response. Responses were graded as negative if the antigen-specific response was <0.35 IU/ml and the mitogen stimulated IFN-γ response was ≥0.5 IU/ml. Responses were indeterminate if either the IFN-γ response in the unstimulated sample was ≥8 IU/ml regardless of the antigen-specific and the mitogen-stimulated responses or if both the antigen-specific response was <0.35 IU/ml and the mitogen-stimulated IFN-γ response was ≤0,5 IU/ml.

### Statistical Analyses

Data were double entered, and all statistical analyses were performed using Stata 11.0 (Stata Corp., Texas, USA). The distribution of continuous variables was assessed for normality. Chi squared and Fisher’s exact tests were used to test for differences in proportions. The association between LTBI and the level of acute phase response was assessed in a linear regression model; the IGRA result (positive vs. negative) was included as independent variable with log-transformed AGP as the outcome variable. The model was further adjusted for age, sex, HIV status and CD4 count. P-values <0.05 were considered significant.

## Results

Among 255 approached household contacts and 199 neighborhood controls, a total of 245 (96%) household contacts and 192 (97%) neighborhood controls had available QFT-IT data and were included in the present analyses. The mean (SD) age was 34.5 (12.8) years, median (IQR) bmi was 21.7 (19.9–24.2) kg/m^2^ and 54% (234) were females.

The overall HIV prevalence was 11% (48) and 92% (402) had visible BCG scar. A summary of the baseline characteristics stratified by household contacts and neighborhood controls are shown in **Table 1**. Among household contacts, the proportion of QFT-IT positive was 59% (145/245), (95% CI: 53–65%) compared to 41% (78/192), (95% CI: 34–48%) in the neighborhood controls (p = 0.001) (**Table 2**). The proportion of indeterminate results among household contacts and neighborhood controls was 7% (16/245), (95% CI: 3.4–9.6%) and 8% (15/192), (95% CI: 4.0–12%) (p = 0.74), respectively. Complete QFT-IT results stratified by household contacts and neighborhood controls are shown in Table 2. The QFT-IT results stratified by HIV status are shown in **Table 3** **.** There was no association between the QFT-IT result and the covariates: sex, age, BCG scar, HIV status, CD4 count, hemoglobin, BMI, and WBCs (data not shown).

**Table 1:** Baseline characteristics of neighborhood controls (n = 192) and household contacts (n = 245) of tuberculosis patients.

	Neighborhood controls		Household contacts	
	N	Mean [SD] or % (n)	N	Mean [SD] or % (n)
Female sex	192	43% (82)	245	62% (152)
Age, years	192	34 [11.99]	245	35 [13.43]
HIV positive	192	9% (17)	245	13% (31)
BCG scar	191	94% (180)	239	93% (222)
CD4 count, cell count/µL, HIV positive individuals	17	574.82 [848.20]	31	376.48 [262.20]
CD4 count, cell count/µL, HIV negative individuals	175	595.58 [318.52]	213#	655.68 [446.80]
Hemoglobin, g/dL	192	14.08 [2.41]	245	13.66 [2.43]
BMI, Kg/M2	192	22.33 [3.74]	244	22.67 [4.11]
Leukocytes, cells/µL	192	4798.96 [1340.70]	245	4977.76 [1642.10]
Neutrofile granulocytes, cells/µL	192	2117.26 [969.22]	245	2307.91 [1097.70]
AGP*, g/L	192	0.68 (0.59–0.85)	0	**–**

Data are mean [SD] or % (n) unless otherwise stated.

* Median (interquartile range).

# One missing CD4 count.

**Table 2:** The QFT-IT results stratified by neighborhood controls (n = 192) and household contacts (n = 245).

	Neighborhood controls		Household contacts	
	N	% (n) [95% CI]	N	% (n) [95% CI]
QFT-IT positive	192	41% (78) [34–48%]	245	59% (145) [53–65%]
QFT-IT negative	192	52% (99) [44–59%]	245	34% (84) [28–40%]
QFT-IT indeterminate	192	8% (15) [4.0–12%]	245	7% (16) [3.4–9.6%]

Data are proportions in % (n) with [95% CI].

**Table 3:** The QFT-IT results stratified by HIV status (n = 437).

	HIV positive		HIV Negative	
	N	% (n) [95% CI]	N	% (n) [95% CI]
QFT-IT positive	48	54% (26) [40–69%]	389	51% (197) [46–56%]
QFT-IT negative	48	35% (17) [22–49%]	389	43% (166) [38–48%]
QFT-IT indeterminate	48	10% (5) [1.7–19%]	389	7% (26) [4.2–9.1%]

Data are proportions in % (n) with [95% CI].

However, in a multiple linear regression model with AGP (log-transformed) as dependent variable and the QFT-IT result being the predictor, the level of AGP was 10% higher (10^B^ 1.10, 95% CI 1.01; 1.20, p = 0.03) among participants with a positive QFT-IT test (suspected LTBI) compared to participants with a negative test (suspected non-LTBI). The regression model was adjusted for sex, age, CD4 and HIV status. The result of the regression model is presented in an online data supplement (Data S1).

## Discussion

These data affirms the fact that LTBI is highly prevalent among urban Tanzanians, also among people with no known exposure or history of TB. Furthermore, our data indicate that LTBI may be associated with subclinical inflammation. In Tanzania, the national HIV prevalence (in 2007) was estimated to be 5.7% [11] while little is known about the present prevalence of LTBI in Tanzania. Corbett E et al estimated in 2000 that 33% of the adult population in Tanzania is infected with *M. tuberculosis* [12]. Based on the results of the QFT-IT, we found that almost half of the neighborhood controls to TB patients were LTBI, despite no known source of TB exposure within the household. A study from the northern part of Tanzania investigated the prevalence of LTBI among pregnant women using the TST and reported a prevalence between 26.2–37.4% [13]. The median age of those screened was 25 years ranging from 16–40 years. In line with this study we found 35. 9% to be QFT-IT positive among women younger than 40 years. Our results are in line with data from a study conducted in Ethiopia, also one of the world’s 22 TB high burden countries, where an LTBI prevalence of 51% was found among healthy blood donors using the QFT-IT [14]. Whether the neighborhood controls are representative of the general population in Mwanza City and Tanzania as a whole can be discussed. Our study design where the neighborhood controls was recruited through a verified TB patient introduce a possibility of bias toward a higher prevalence of LTBI since we do not have any estimates regarding the zone of influence a TB case may exercise on the community. A previous study has pointed out that a TB case might influence the incidence of TB up to 10th household in either direction [15]. In our study the neighborhood controls were living within a distance of 10–20 houses of a known TB patient. Although we used confirmed TB patients to localize household contacts and neighborhood controls we did not perform QFT-IT on the exact TB patient giving rise to the following household contact and neighborhood controls. However a previous study [16] likewise nested within the framework of the two nutrition studies among TB patients [9], [10] found 74% to be QFT-IT positive. The positive proportion of 41% LTBI in an urban setting of Mwanza, the 2^nd^ largest city in Tanzania, implies that TB transmission is certainly taking place in this region. The fact that the four different TB clinics which participated in the study ensured that most of the city area was covered and we believe that our findings are valid as a crude estimate of the prevalence of LTBI in Mwanza city.

Preventive treatment of LTBI with isoniazid has been shown to reduce the risk of future disease by 75%–90% [17] and a positive TST has proven useful to identify individuals who benefits from isoniazid treatment [2]. Although this finding is not yet proven for QFT-IT it is likely that the same applies for QFT-IT positive individuals [2]. Among household contacts we found nearly 60% to be QFT-IT positive and 13% to be HIV positive. It is not surprising to find a higher prevalence of QFT-IT positives in this population and cost effectiveness studies of systematic testing and treatment of household contacts are clearly warranted.

Whether the IGRAs should replace the TST in low- and middle-income countries for the diagnostic of LTBI has not been within the study objectives to determine. WHO strongly discourages the use of IGRAs for the diagnostics of LTBI in low- and middle-income countries [18] partly due to insufficient data on the performance of IGRAs and partly due to increased cost of IGRAs compared to TST. We do not find that our study has the strength to challenge this policy statement. However, our study confirms previous findings that prior BCG vaccination do not affect the QFT-IT result [19] and we also did not find any association between age and LTBI. However we found an association between LTBI and elevated levels of the acute phase protein AGP among otherwise healthy neighborhood controls, which to our knowledge has not been reported previously. AGP is synthesized in the liver [7] and involved in the induction of non-specific resistance to infection [20], [21]. AGP levels are elevated in patients with active TB [7] and it may be a possible marker of slow response to anti-TB treatment [8]. The elevated AGP levels found among neighborhood controls with LTBI warrant further studies. We need to assess whether people with LTBI and elevated AGP are in fact active TB in an early phase, or whether LTBI may cause chronic low grade inflammation. Likewise further studies are needed in order to determine whether living with elevated markers of inflammation has any implications on the general health status. Low-grade inflammation plays a role in the pathogenesis of diabetes mellitus type II [22], and since diabetes is known to be associated with TB [23], [24], this may be one of several links between the two diseases.

### Conclusion

LTBI is highly prevalent among apparently healthy urban Tanzanians even without known exposure to TB in the household. LTBI was found to be associated with elevated serum levels of AGP. This may reflect a state of low-grade inflammation and could represent a possible link between diabetes mellitus type II and TB.

## Supporting Information

Data S1 (DOCX)
