# Supplementary material for: The Prevalence of Latent Mycobacterium tuberculosis Infection Based on an Interferon-γ Release Assay: A Cross-Sectional Survey among Urban Adults in Mwanza, Tanzania
Source: PLoS One. 2013 May 21;8(5):e64008. doi: 10.1371/journal.pone.0064008 (PMC3660306; doi:10.1371/journal.pone.0064008)
Supplement: Data S1 — (DOCX) [file pone.0064008.s001.docx]

Online supplement as requested by reviewers :

| Results from the multiple linear regression model | | | | |
| --- | --- | --- | --- | --- |
|  | 10^B | P-value | (95 % CI) |  |
| QFT-IT positive vs. QFT-IT negative | 1,1035016833552 | 0,03 | (1.01-1.20) |  |
| QFT-IT indeterminate vs. QFT-IT negative | 1,11169460997613 | 0,19 | (0.95- 1.30) |  |
| age, years | 1,00079240305754 | 0,65 | (1.00-1.004) |  |
| male sex | 0,929893046110536 | 0,09 | (0.86- 1.01) |  |
| HIV positive vs. HIV negative | 1,28090316866734 | 0,001 | (1,11-1,48) |  |
| CD4 count, cell count/μL | 1,00003177617914 | 0,55 | (1.00- 1,0001) |  |
